# Synthesis of BiOX-Red Mud/Granulated Blast Furnace Slag Geopolymer Microspheres for Photocatalytic Degradation of Formaldehyde

**DOI:** 10.3390/ma17071585

**Published:** 2024-03-30

**Authors:** Ping Lu, Na Zhang, Ying Wang, Yidi Wang, Jiale Zhang, Qingyi Cai, Yihe Zhang

**Affiliations:** 1 Engineering Research Center of Ministry of Education for Geological Carbon Storage and Low Carbon Utilization of Resources, China University of Geosciences, Beijing 100083, China; 2 Beijing Key Laboratory of Materials Utilization of Nonmetallic Minerals and Solid Wastes, China University of Geosciences, Beijing 100083, China; 3 National Laboratory of Mineral Materials, China University of Geosciences, Beijing 100083, China; 4 School of Materials Science and Technology, China University of Geosciences, Beijing 100083, China

**Keywords:** red mud, granulated blast furnace slag, geopolymer microspheres, photocatalysis, formaldehyde

## Abstract

Release of formaldehyde gas indoors is a serious threat to human health. The traditional adsorption method is not stable enough for formaldehyde removal. Photocatalytic degradation of formaldehyde is effective and rapid, but photocatalysts are generally expensive and not easy to recycle. In this paper, geopolymer microspheres were applied as matrix materials for photocatalysts loading to degrade formaldehyde. Geopolymer microspheres were prepared from red mud and granulated blast furnace slag as raw materials by alkali activation. When the red mud doping was 50%, the concentration of NaOH solution was 6 mol/L, and the additive amount was 30 mL, the prepared geopolymer microspheres possessed good morphological characteristics and a large specific surface area of 38.80 m^2^/g. With the loading of BiOX (X = Cl, Br, I) photocatalysts on the surface of geopolymer microspheres, 85.71% of formaldehyde gas were adsorbed within 60 min. The formaldehyde degradation rate of the geopolymer microspheres loaded with BiOI reached 87.46% within 180 min, which was 23.07% higher than that of the microspheres loaded with BiOBr, and 50.50% higher than that of the microspheres loaded with BiOCl. While ensuring the efficient degradation of formaldehyde, the BiOX (X = Cl, Br, I)-loaded geopolymer microspheres are easy to recycle and can save space. This work not only promotes the resource utilization of red mud and granulated blast furnace slag, but also provides a new idea on the formation of catalysts in the process of photocatalytic degradation of formaldehyde.

## 1. Introduction

People spend most of their time working, studying and living indoors, so indoor air quality can directly affect human health. Formaldehyde, as one of the volatile organic contaminants (VOCs), is a typical harmful gas produced during house decoration [1]. It can cause great damage to various aspects of the human body, such as the respiratory tract, immune system and skin, and can even lead to cancer [2]. Therefore, the methods of formaldehyde detection and removal have attracted extensive attention from governments and scholars. Currently, the main ways to deal with formaldehyde are physical adsorption, photocatalytic degradation and biodegradation [3,4]. The traditional adsorption technology has a low adsorption capacity, and this shortcoming is especially obvious when the concentration of formaldehyde is low [5]. Photocatalytic degradation is a cleaner technology, which can degrade formaldehyde into CO_2_ and H_2_O, and the removal effect of formaldehyde is more stable [6,7,8]. Yang et al. [9] successfully constructed a photocatalytic system that can produce H_2_ and degrade formaldehyde at the same time. Yuan et al. [10] proposed a “pizza”-like composite, which is highly effective in mineralizing formaldehyde gas under visible light. Liu et al. [11] found that, after the addition of diatomite, the reactions of the photocatalysts can be improved, which can degrade formaldehyde more efficiently.

Red mud is a by-product released during the production of alumina in the aluminum industry [12]. According to statistics, 1–2 tons of red mud are released for every ton of alumina produced [13]. Red mud has a high alkalinity, fine particles and some harmful metals [14]. However, in the case of such a large amount of red mud emissions, its utilization rate is low [15] at less than 10%. A large amount of red mud cannot be reasonably utilized, but can only be piled up and stored, which will occupy a large amount of land resources in the long run [16]. Red mud is currently used mainly in the field of construction and building materials [17,18], the recovery of valuable metals [19,20], and other areas [21,22]. The addition of red mud can effectively improve the compressive strength of the system [23] and the microstructure will be denser [24]. Concrete with red mud also meets safety and toxicity standards and will not have negative effects [25]. Attempts have been made to prepare red mud as a catalyst after modification treatment for the degradation of levofloxacin [26] and sulfamethoxazole [27], both of which could achieve more than 90% degradation within 1 h. Red mud and paper ash were used to prepare a composite material [28], which can adsorb toxic and harmful elements in the soil, thus achieving the purpose of soil remediation. Li et al. [29] showed that, after the addition of red mud, it can form a new phase that is favorable for the solidification of heavy metals, and reduce the leaching of a variety of heavy metals in fly ash. In addition, red mud can also adsorb a variety of other heavy metal ions [30,31,32]. The coupling of red mud and phosphorus mud [33] can be utilized to absorb NO_x_ and avoid secondary pollution.

Currently, photocatalysts are mainly in the form of powder, which does not conveniently allow solid–liquid separation after processing pollutants, limiting their use [34]. Loading photocatalysts onto matrix materials can effectively solve this problem. Geopolymer microspheres can be prepared as geopolymers can be cured quickly under certain conditions [35], which is a cheap and environmentally friendly matrix material. The adsorption of F^−^ in water could reach 121.77 mg/g after loading CeO_2_ or Fe_2_O_3_ [35,36]. Meanwhile, the modified microspheres showed good adsorption effects on heavy metal ions such as As(III), As(V), and Ni(II) [37,38]. Geopolymer microspheres have been studied a lot for the removal of F^−^ as well as heavy metals [39] in water, but little research has been conducted on their use as matrix materials loaded with photocatalysts for the degradation of gas pollutants.

Both red mud and granulated blast furnace slag are typical solid wastes with alkaline activity [40,41], from which silicon and aluminum ions can be dissolved in alkaline solution, thus possessing certain cementitious activity [42,43]. In this work, red mud and granulated blast furnace slag were alkali activated to prepare geopolymer microspheres, which were used as the matrix materials to load BiOX (X = Cl, Br, I) photocatalyst for formaldehyde degradation. The effects of red mud dosing, NaOH concentration, and NaOH addition on sphericity of the geopolymer microspheres were investigated. Furthermore, the differences in photocatalytic degradation performance of formaldehyde by microspheres loaded with BiOX (X = Cl, Br, I) were compared and analyzed. The aim of this work was to successfully prepare inexpensive, easy-to-recycle and well-formed matrix materials for photocatalyst loading, while effectively utilizing industrial solid wastes, i.e., red mud and granulated blast furnace slag. In addition, the BiOX-loaded microsphere composites can efficiently degrade formaldehyde via photocatalysis. This work provides a method to prepare photocatalytic composite materials using red mud, granulated blast furnace slag and BiOX (X = Cl, Br, I), which can effectively solve the indoor air pollution problem and promote solid waste utilization.

## 2. Experimental Method

### 2.1. Raw Materials

The red mud used in the experiment was provided by Aluminum Corporation of China Limited Zhongzhou Branch, Jiaozuo, Henan, China. The granulated blast furnace slag was supplied by Henan Yuanheng Environmental Protection Engineering Co., Ltd., Gongyi, Henan, China. The NaOH (analytical purity) and polyethylene glycol 2000 (chemically pure) was sourced from Yili Fine Chemicals Co., Ltd., Beijing, China. The KX (X = Cl, Br, I) (analytical purity) and formaldehyde solution were from Tianjin Zhiyuan Chemical Reagent Co., Tianjin, China. The BiNO_3_·5 H_2_O (analytical purity) was from Xilong Science Co. Ltd., Shantou, Guangdong, China.

**Figure 1:**
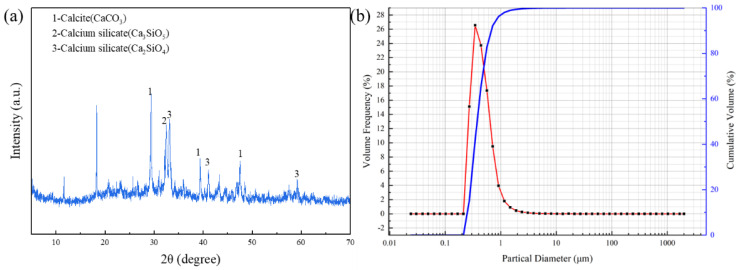
XRD pattern (**a**) and particle diameter distribution (**b**) of red mud.

Figure 1a shows the XRD pattern of the raw red mud, which indicates that the main components are CaCO_3_, Ca_2_SiO_4_, and Ca_3_SiO_5_. Figure 1b shows the particle diameter distribution of the red mud, in which the D50 is 0.374 μm, and the D90 is 0.661 μm. Table 1 shows the XRF test results of the red mud, of which the CaO accounted for a relatively large proportion (42.99%); this was followed by SiO_2_, Al_2_O_3_, Fe_2_O_3_, etc.

**Figure 2:**
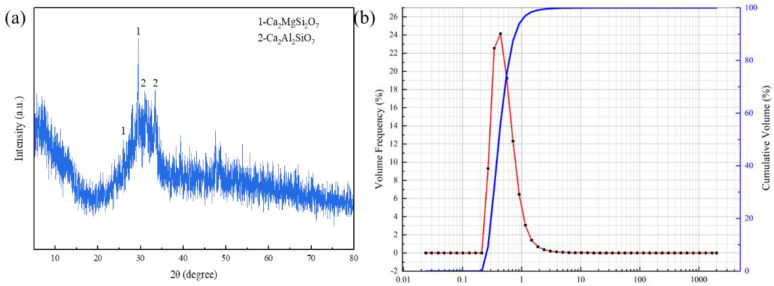
XRD pattern (**a**) and particle diameter distribution (**b**) of granulated blast furnace slag.

Figure 2a shows the XRD pattern of granulated blast furnace slag, which has a diffuse peak at about 30°, indicating that the granulated blast furnace slag has a relatively low crystallinity. Figure 2b is the particle size distribution of the granulated blast furnace slag, whose D50 is 0.413 μm and D90 is 0.772 μm, which is slightly higher than that of red mud. Table 1 also shows the XRF test results of granulated blast furnace slag, its main component is still CaO, accounting for 42.76%, followed by SiO_2_, Al_2_O_3_, etc.; the Fe_2_O_3_ content in red mud varies widely.

### 2.2. Characterization Methods

The mineralogical components of microspheres were analyzed via X-ray powder diffraction (XRD) (Bruker D8 Advance Instrument, Bruker Corporation, Karlsruhe, Germany) using Cu Kα radiation (λ = 1.54056 Å) at 40 kV and 40 mA. The scanning range was 5°~80° and the scanning speed was 5°/min. The RGGMs was ground into powder for XRD, FTIR and UV-Vis tests. The particle size of red mud and granulated blast furnace slag powder was tested using a laser particle size analyzer (Bettersize2000, Dandong Baxter Instrument Co., Ltd., Dandong, China), where sodium hexametaphosphate was used as a dispersant. Fourier-transform infrared (FTIR) spectra were recorded using a Nicolet IS10 spectrometer (Nicolet, Mountain, WI, USA) within the scan range of 4000–550 cm^−1^. A total of 1.5 mg of RGGMs and 70 mg of KBr were mixed well and pressed into tablets for FTIR testing. The specific surface area of the microsphere was determined via the Brunauer–Emmett–Teller (BET) method with an ASAP 2010 porosimeter. XPS data were collected using an ESCALAB 250 Xi photoelectron spectrometer (Thermo Fisher Scientific, Waltham, MA, USA) equipped with a monochromatic Al Kα radiation source. The microscopic morphology of the samples was observed and analyzed using an SU 8020 field emission scanning electron microscope (SU 8020, Hitachi Ltd., Tokyo, Japan). The microspheres were placed on the conductive tape and it was ensured that they would not be blown off; they were then sprayed with platinum for SEM testing. Electrochemical analysis tests, including Mott–Schottky (M–S) plots, photocurrent response, and electrochemical impedance spectroscopy (EIS) of samples, were performed on a CHI660D instrument (CH Instruments, Beijing, China). A conductive substrate coated with photocatalyst was used as the working electrode, a platinum sheet as the counter electrode, Ag/AgCl as the reference electrode, and Na_2_SO_4_ as the electrolyte in order to construct a three-electrode system for electrochemical testing. Absorption spectra were obtained using a UV-3600i Plus spectrometer (Shimadzu, Kyoto, Japan).

### 2.3. Preparation of RGGMs and BiOX (X = Cl, Br, I)-RGGMs

**Figure 3:**
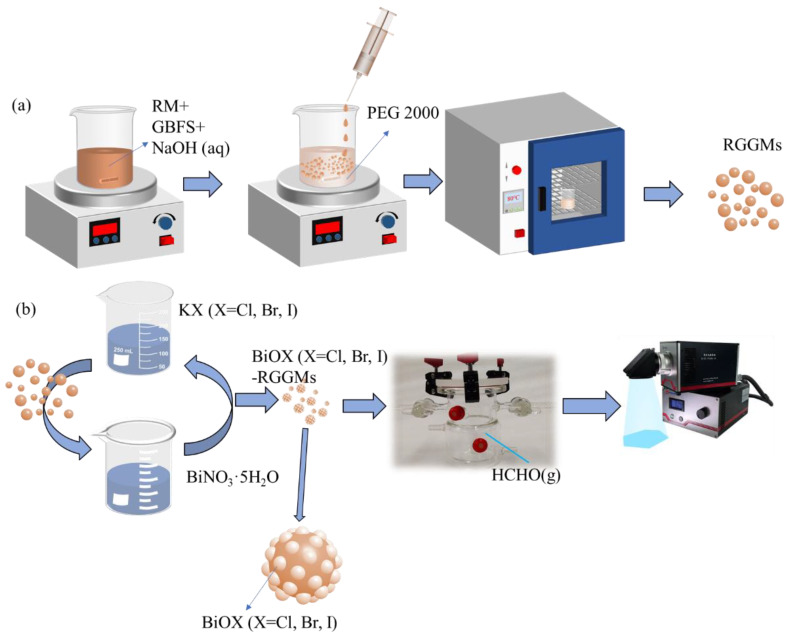
Preparation of RGGMs (**a**) and photocatalytic degradation of formaldehyde after loading BiOX (X = Cl, Br, I) (**b**).

The preparation of red mud/granulated blast furnace slag geopolymer microspheres (RGGMs) and the loading of BiOX (X = Cl, Br, I) are shown in Figure 3a,b. The photocatalyst-loaded microspheres were defined as BiOX (X = Cl, Br, I)-red mud/slag geopolymer microspheres (BiOX (X = Cl, Br, I)-RGGMs).

Firstly, red mud and granulated blast furnace slag powder were added into NaOH solution and stirred for 5 min. The slurry was sucked into a syringe and evenly dripped into polyethylene glycol 2000 (PEG2000), which is a dispersant. Under the effect of the shear force and surface tension of PEG2000, the slurry can form microspheres rapidly when added to PEG2000. After that, the beaker was placed into an oven at 80 °C for 3 h. The RGGMs were obtained after filtration, washing and drying. The prepared RGGMs were alternately immersed in KX (X = Cl, Br, I) and Bi(NO_3_)_3_ 5H_2_O solutions and this was repeated 20 times. Then the microspheres were dried in an oven at 80 °C to obtain BiOX (X = Cl, Br, I)-RGGMs.

### 2.4. Photocatalytic Performance Evaluation

The photocatalytic performance of the obtained samples was evaluated by degrading formaldehyde on the basis of obtaining the optimal process for the preparation of RGGMs. The light source was a 300 w Xe lamp. A pump suction formaldehyde tester was used to measure the concentration of formaldehyde gas in the container. The concentration of formaldehyde in the container during the adsorption process was measured every 10 min, 4 mL of gas was extracted and the pattern was measured 6 times. The concentration of formaldehyde remaining in the container during degradation was measured every 30 min, 4 mL of gas was extracted, and the pattern was also measured 6 times.

## 3. Results and Discussion

### 3.1. Synthesis and Microscopic Morphology of RGGMs

When synthesizing RGGMs, the sphericity of RGGMs is affected by various factors. In this experiment, the effects of red mud dosing, NaOH concentration, and liquid–solid ratio on the sphericity of RGGMs were investigated.

#### 3.1.1. Effect of Red Mud Dosing on the Sphericity of Microspheres

**Figure 4:**
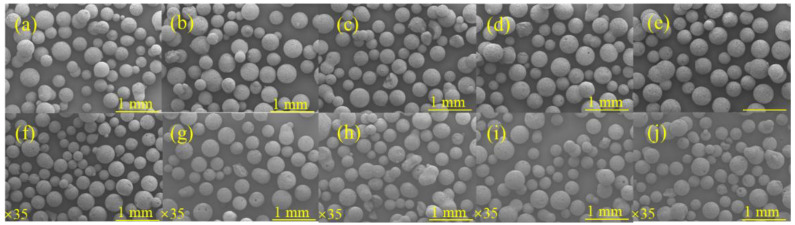
Microscopic morphology of RGGMs with 10% (**a**), 20% (**b**), 30% (**c**), 40% (**d**), 50% (**e**), 60% (**f**), 70% (**g**), 80% (**h**), 90% (**i**), 100% (**j**) red mud dosage.

Figure 4a–j show the change of sphericity of microspheres under different red mud dosage. In the case of less red mud doping, the powder has high activity after alkali excitation. It is very easy to be agglomerated together during maintenance, and the balling rate is not high. In the case of more red mud doping, the powder is less active after alkali excitation, and the ball formation is poor. It is especially obvious at 80–100% red mud doping, and there are more incomplete microspheres. To comprehensively analyze the actual balling situation and the difference in sphericity, a group with 50% red mud doping was selected for the following experiments.

#### 3.1.2. Effect of NaOH Concentration on the Sphericity of Microspheres

**Figure 5:**
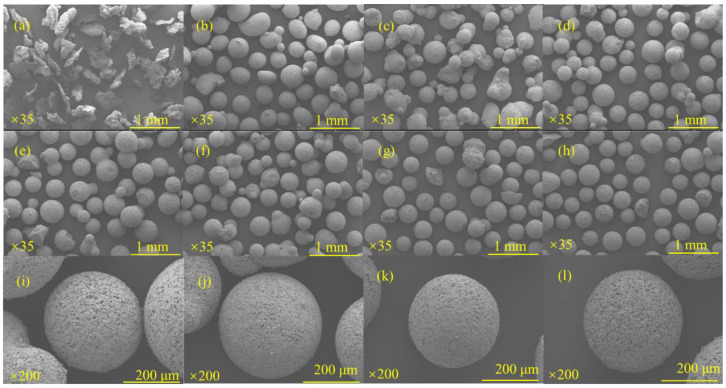
Microscopic morphology of RGGMs at 1 mol/L (**a**), 2 mol/L (**b**), 3 mol/L (**c**), 4 mol/L (**d**), 5 mol/L (**e**), 6 mol/L (**f**), 7 mol/L (**g**), 8 mol/L (**h**) NaOH and enlarged images of RGGMs at 5 mol/L (**i**), 6 mol/L (**j**), 7 mol/L (**k**), 8 mol/L (**l**).

Figure 5a–l show the differences in microsphere formation and sphericity under different NaOH solutions. It can be seen that at lower concentrations of NaOH solution, most of the samples obtained were flaky, incomplete, and easily adhered together, which may be due to the fact that the activity of the powders had not been fully activated. When the concentration of NaOH solution reached 5 mol/L or more, the overall sphere formation was good, so the spheres were further enlarged to investigate the integrity of their surfaces. When the concentration of NaOH solution was changed from 5 mol/L to 6 mol/L, the sphericity became better, but with the further increase of concentration, more gaps and holes appeared on the surface of the sphere, and there was a certain decrease in the sphericity. Therefore, for the following synthesis process, the concentration of NaOH solution was chosen to be 6 mol/L.

#### 3.1.3. Effect of Liquid–Solid Ratio on the Sphericity of Microspheres

**Figure 6:**
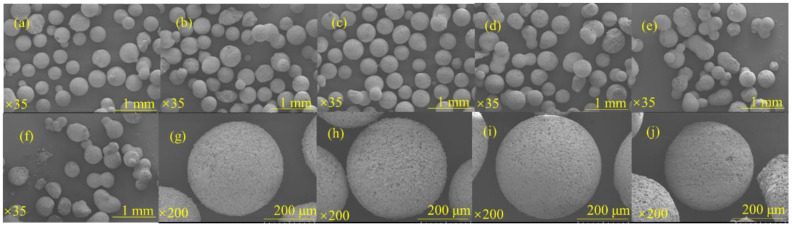
Microscopic morphology of RGGMs under 20 mL (**a**), 25 mL (**b**), 30 mL (**c**), 35 mL (**d**), 40 mL (**e**), 45 mL (**f**),NaOH solution additions and enlarged images of RGGMs under 20 mL (**g**), 25 mL (**h**), 30 mL (**i**), 35 mL (**j**).

Figure 6a–j show the change of sphericity of microspheres under different NaOH solution additions. When the NaOH solution is added in small amounts, the overall alkalinity of the system is low, and some of the slurry is difficult to form a ball. However, after more NaOH solution is added, the liquid–solid ratio of the system is too high, and there is too much water in the system, which is not conducive to the formation of microspheres. Further observing the groups with better microsphere formation, it is easy to notice that the best microsphere formation was achieved at a NaOH solution addition of 30 mL (liquid-solid ratio of 2).

Therefore, the optimal experimental conditions were as follows: 50% red mud doping, 6 mol/L NaOH solution concentration, and 30 mL NaOH solution addition. This ratio was used in all of the following experimental studies.

### 3.2. Mineral Composition Analysis of RGGMs

**Figure 7:**
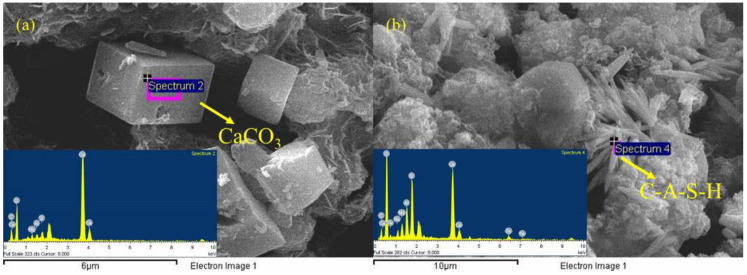
CaCO_3_ (**a**) and C-A-S-H (**b**) in RGGMs.

In order to further analyze the mineral composition of the RGGMs, some areas on the surface of the microspheres were selected for magnified observation and energy spectrum scanning. As shown in Figure 7a,b, the surface of RGGMs has relatively more cubic lumpy and needle-like materials. By analyzing the EDS energy spectrum scanning results in Table 2, the Ca/C in the cubic bulk material region is close to 1, and the O/C is close to 3, presuming that the cubic bulk material is CaCO_3_. The content of Si and Al elements in the needle-like material region is higher, presuming that the generated material is the calcium aluminum silicate hydroxide (C-A-S-H) with small amounts of Mg, Al, and Fe elements [44], which is a common product in alkali-activated cementitious materials to improve the strength of the system [40].

**Figure 8:**
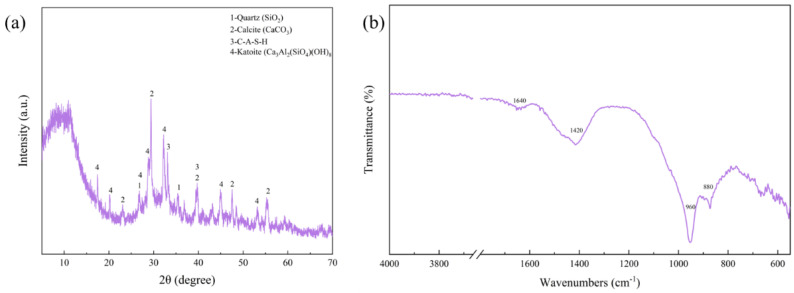
XRD pattern (**a**) and infrared spectra (**b**) of RGGMs.

Figure 8a shows the XRD patterns of the RGGMs, which can further prove the above presumption that the generated cubic mass is CaCO_3_ [18] and the needle-like generated material is C-A-S-H. By analyzing the FT-IR spectra of the RGGMs in Figure 8b, it can be seen that the main vibration peaks of the microspheres are at about 880 cm^−1^, 960 cm^−1^, 1420 cm^−1^, and 1640 cm^−1^; and the absorption band at 1640 cm^−1^ is related to the bending vibration of the H-O-H group that combines with water [45]. The absorption peak at 1420 cm^−1^ is caused by the O-C-O bond vibration in ${CO}_{3}^{2-}$, which may be related to the carbonization of part of Ca(OH)_2_ by CO_2_. The absorption bands at around 960 cm^−1^ and 880 cm^−1^ may be the stretching vibrational bands of Si-O in the [SiO_4_]^4−^ tetrahedra of the C-A-S-H. This is consistent with the results of XRD and SEM analyses, indicating that CaCO_3_ as well as C-A-S-H are truly existent in RGGMs.

**Figure 9:**
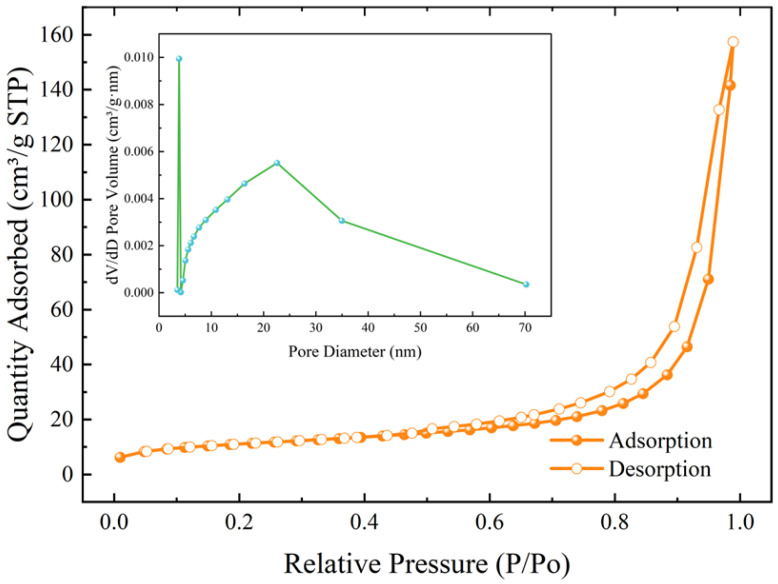
N_2_ adsorption–desorption curves and pore size distribution of RGGMs.

Figure 9 shows the N_2_ adsorption–desorption curves and pore size distributions of the microspheres. The RGGMs showed a type IV curve (H3 hysteresis loop), indicating the existence of predominantly mesoporous structures in the microspheres [46]. The BJH desorption test showed that the average pore size of the RGGMs was 21.99 nm. The large specific surface area allows the microspheres to be used as carriers for photocatalyst loading, which can broaden the application areas [47].

### 3.3. HCHO Removal Performance of BiOX (X = Cl, Br, I)-RGGMs

#### 3.3.1. Loading of BiOX (X = Cl, Br, I)

**Figure 10:**
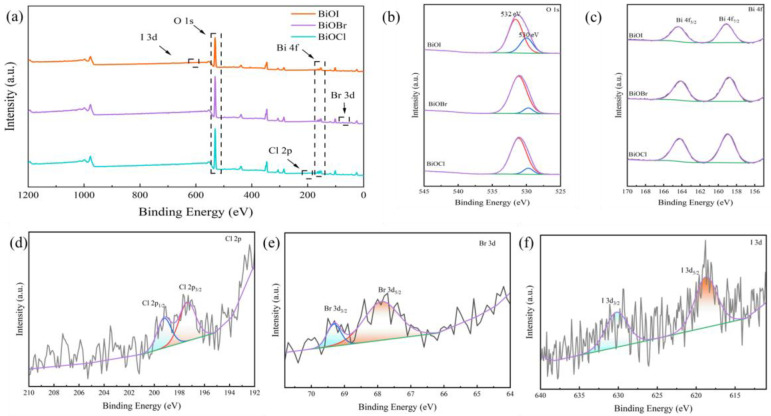
XPS spectra of BiOX (X = Cl, Br, I)-RGGMs: (**a**) full spectrum, (**b**) O 1s, (**c**) Bi 4f, (**d**) Cl 2p, (**e**) Br 3d and (**f**) I 3d.

Figure 10a–f show the XPS spectra of BiOX (X = Cl, Br, I)-RGGMs, in which the peaks in the total spectrum are not obvious enough due to the low loading of BiOX (X = Cl, Br, I). Under high-resolution XPS, the peaks at around 530 eV and 532 eV in Figure 10b correspond to O^2−^ in the Bi-O and O-Si-O bonds, respectively, and the peaks at around 159 and 165 eV in Figure 10c belong to Bi 4f_7/2_ and Bi 4f_5/2_, which indicate the presence of Bi^3+^ [48]. The peaks at about 197 eV and 199 eV in Figure 10d–f belong to Cl 2p_3/2_ and Cl 2p_1/2_, 68 eV and 69 eV belong to Br 3d_5/2_, Br 3d_3/2_, 619 eV and 630 eV belong to I 3d_5/2_ and I 3d_3/2_, respectively.

**Figure 11:**
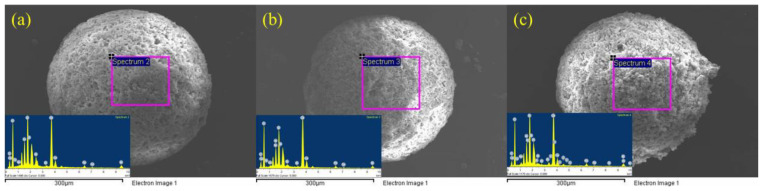
Microscopic morphology of microspheres after loading BiOCl (**a**), BiOBr (**b**) and BiOI (**c**).

Figure 11a–c are the microscopic morphology picture of the microspheres after loading the photocatalysts, and Table 3 is the corresponding SEM-EDS scanning results. After loading BiOX (X = Cl, Br, I), the surface of microspheres becomes rough. The energy spectrum scans also contain Bi elements, and the relative contents are all less than 1%. Combined with the XPS results, this altogether proves that BiOX (X = Cl, Br, I) has been successfully loaded onto the microsphere surface.

#### 3.3.2. HCHO Removal Performance

**Figure 12:**
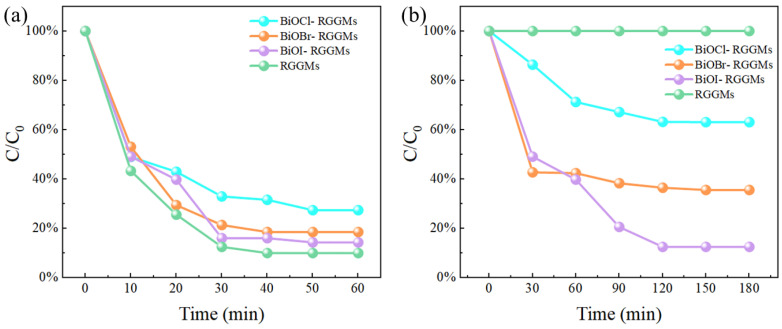
Adsorption (**a**) and degradation (**b**) results of formaldehyde by BiOX (X = Cl, Br, I)-RGGMs and RGGMs.

Figure 12a,b show the results of adsorption and degradation of formaldehyde gas by microspheres before and after loading with BiOX (X = Cl, Br, I). The RGGMs had the best adsorption performance for formaldehyde when not loaded with any photocatalyst, with an adsorption rate of 89.97%. The overall difference in the adsorption of formaldehyde gas by microspheres after loading different types of photocatalysts was not too considerable. BiOI-RGGMs had the best adsorption effect with 85.71% adsorption rate, which was 13.09% higher than that of BiOCl-RGGMs. These differences could be attributed to the fact that some of the pores on the surface of RGGMs were closed after loading with BiOX (X = Cl, Br, I), resulting in a decrease in the adsorption rate when loaded with photocatalysts. When degrading formaldehyde, the microspheres loaded with different photocatalysts all degraded formaldehyde faster in the first 1 h. After the degradation time reached 1.5 h, the degradation rate tended to level off, and the maximum degradation rate was reached at about 2 h. BiOI-RGGMs had the best degradation effect on formaldehyde gas, which was able to reach 87.46%, followed by BiOBr-RGGMs and BiOCl-RGGMs, with degradation rates of 64.39% and 36.96%, respectively, at 3 h. In addition, RGGMs did not show any degradation effect on formaldehyde, which indicates that all the degradation performance of BiOX (X = Cl, Br, I)-RGGMs on formaldehyde can be traced to their surface-loaded BiOX (X = Cl, Br, I).

After loading a low amount of photocatalyst, it still has a relatively good formaldehyde removal effect. This indicates that BiOX (X = Cl, Br, I)-RGGMs are good matrix materials, and the photocatalysts loaded on their surfaces can be fully exposed to light to achieve good photocatalytic effects.

### 3.4. Optoelectronic Properties of BiOX (X = Cl, Br, I)-RGGMs

**Figure 13:**
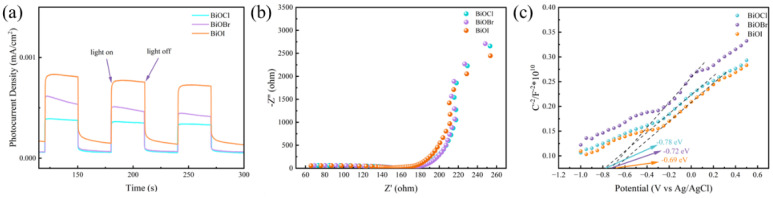
Photocurrent response (**a**), EIS curve (**b**), M-S curve of BiOX (X = Cl, Br, I)-RGGMs (**c**).

Figure 13a shows the photocurrent density profile of BiOX (X = Cl, Br, I)-RGGMs. BiOI-RGGMs provide the highest photocurrent response with the best charge separation efficiency, followed by BiOBr-RGGMs and BiOCl-RGGMs. The EIS spectra and Mott-Schottky curves of BiOX (X = Cl, Br, I)-RGGMs are shown in Figure 13b,c, respectively. The diameter size [49] and photocurrent density of BiOX (X = Cl, Br, I)-RGGMs are consistent with each other, which indicates that the charge separation efficiency follows the order of BiOI-RGGMs > BiOBr-RGGMs > BiOCl-RGGMs. The slope of the MS curve is >0, indicating that BiOX (X = Cl, Br, I) is an n-type semiconductor [50].

**Figure 14:**
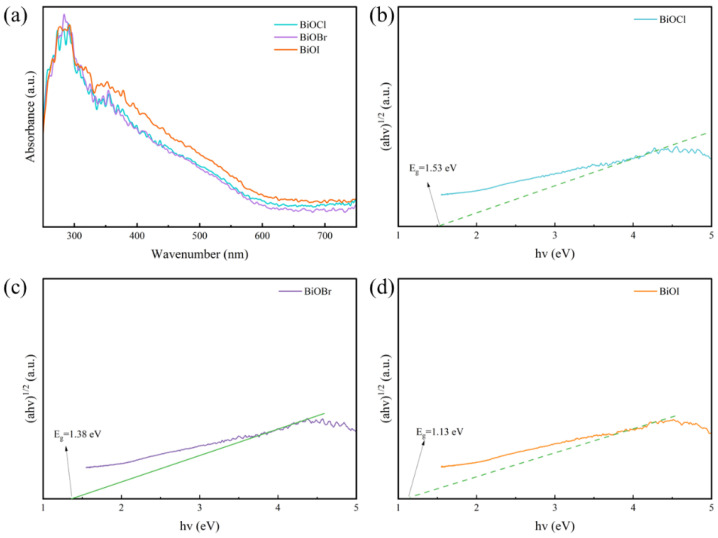
UV-vis absorption spectra of BiOX (X = Cl, Br, I)-RGGMs (**a**) and the bandgap value of BiOCl-RGGMs (**b**), BiOBr-RGGMs (**c**) and BiOI-RGGMs (**d**).

Figure 14a–d show the UV-Vis pattern of BiOX (X = Cl, Br, I)-RGGMs. According to the equation *αhν = A(hν − Eg)^1/2^*, where *α*, *h*, *ν* and A represent the photoabsorption coefficient, the Planck constant, the light frequency and a constant, respectively, the energy band gaps of BiOX (X = Cl, Br, I)-RGGMs are 1.53 eV, 1.38 eV, and 1.13 eV, respectively. It can also be concluded that BiOI-RGGMs have the smallest energy band gap and the largest light absorption range, followed by BiOBr-RGGMs and BiOCl-RGGMs.

Table 4 shows a summary of the formaldehyde degradation performance of the other photocatalyst composites. In comparison, BiOX (X = Cl, Br, I)-RGGMs could achieve 87.46% degradation rate at 2 h, exhibiting a relatively good performance of photocatalytic degradation of formaldehyde.

## 4. Conclusions

This work focuses on the synthesis of BiOX (X = Cl, Br, I)-RGGMs and their performance in the photocatalytic degradation of formaldehyde. The main conclusions are as follows:

(1) The microspheres had the best sphericity at 50% addition of red mud, 6 mol NaOH solution concentration and 30 mL addition. The main mineral compositions in the microspheres were CaCO_3_ and C-A-S-H. The specific surface area was up to 38.80 m^2^/g, which was mainly mesoporous.
(2) After loading BiOX (X = Cl, Br, I), BiOX (X = Cl, Br, I)-RGGMs showed good adsorption and degradation of formaldehyde gas. Among them, BiOI-RGGMs showed the best performance, and the degradation rate of 87.46% could be achieved at about 2 h, followed by BiOBr-RGGMs and BiOCl-RGGMs. It can be found by SEM, TEM and XPS that BiOX (X = Cl, Br, I) has been successfully loaded onto the surface of the microspheres and the difference in the performance of BiOX (X = Cl, Br, I)-RGGMs for the photocatalytic degradation of formaldehyde is in accordance with the results of their photoelectric tests.
(3) BiOX (X = Cl, Br, I)-RGGMs are simple to prepare, inexpensive, easy to recycle and reuse, and have good application prospects in degrading indoor formaldehyde gas. The performance of BiOX (X = Cl, Br, I)-RGGMs in degrading other pollutants deserves further investigation in the future.

## Figures and Tables

**Table 1:** Chemical composition of red mud and granulated blast furnace slag.

Chemical Composition (wt%)	CaO	SiO_2_	Al_2_O_3_	Fe_2_O_3_	SO_3_	Na_2_O	MgO	Others
Red mud	42.99	22.20	10.40	9.89	3.75	3.46	1.76	5.55
Granulated blast furnace slag	42.76	24.85	15.61	0.36	2.64	0.55	7.78	5.45

**Table 2:** SEM-EDS results of RGGMs.

Atomic%	C	O	Na	Mg	Al	Si	Ca	Fe
Figure 7a	17.05	66.22	-	0.85	0.46	0.96	14.46	-
Figure 7b	12.80	69.31	1.23	1.55	2.71	5.21	6.57	0.62

**Table 3:** SEM-EDS results of RGGMs loaded with different photocatalysts.

Atomic%	BiOCl	BiOBr	BiOI
C	17.28	16.34	13.03
O	62.68	64.76	64.19
Na	0.38	0.27	0.75
Al	3.29	3.32	3.20
Si	7.15	5.81	7.13
Ca	8.01	8.80	9.29
Fe	0.60	0.60	0.66
Bi	0.60	0.11	0.30

**Table 4:** Summary of formaldehyde degradation by other photocatalyst composites.

Materials	Lighting Time	Degradation	References
Cu–TiO_2_/wood composites	2 h	85.59%	[51]
Co_3_O_4_/CN-x%CeO_2_ (x = 0, 3, 6) composites	9 h	91.5%	[52]
Tourmaline-titanium dioxide composite	10 h	92.2%	[53]
BiOCl/clinoptilolite composite photocatalyst	2 h	87.7%	[54]

## Data Availability

Data are contained within the article.
